# Gene expression of the endocannabinoid system in endometrium through menstrual cycle

**DOI:** 10.1038/s41598-022-13488-4

**Published:** 2022-06-07

**Authors:** Keisuke Tanaka, Akwasi A. Amoako, Sally Mortlock, Peter A. W. Rogers, Sarah J. Holdsworth-Carson, Jacqueline F. Donoghue, Wan Tinn Teh, Grant W. Montgomery, Brett McKinnon

**Affiliations:** 1 Department of Obstetrics and Gynaecology, The Royal Brisbane and Women’s Hospital, Herston, QLD 4029 Australia; 2 Faculty of Medicine, University of Queensland, Herston, QLD 4006 Australia; 3 Institute for Molecular Bioscience, University of Queensland, St Lucia, QLD 4072 Australia; 4 Department of Obstetrics and Gynaecology, University of Melbourne, Parkville, VIC 3052 Australia; 5 Gynaecology Research Centre, Royal Women’s Hospital, Parkville, VIC 3052 Australia; 6 Julia Argyrou Endometriosis Centre, Epworth HealthCare, Richmond, VIC 3121 Australia; 7 Department of Biomedical Research, University of Berne, Murtenstrasse 35, 3010 Berne, Switzerland

**Keywords:** Physiology, Molecular medicine

## Abstract

Endocannabinoids mediate cellular functions and their activity is controlled by a complex system of enzymes, membrane receptors and transport molecules. Endocannabinoids are present in endometrium, a cyclical regenerative tissue requiring tightly regulated cellular mechanisms for maturation. The objective of this study was to investigate the gene expression of key elements involved in the endocannabinoid system across the menstrual cycle. RNA was isolated from endometrial tissue and genome-wide gene expression datasets were generated using RNA-sequencing. An a priori set of 70 genes associated with endocannabinoid system were selected from published literature. Gene expression across the menstrual cycle was analyzed using a moderated *t* test, corrected for multiple testing with Bonferroni’s method. A total of 40 of the 70 genes were present in > 90% of the samples, and significant differential gene expression identified for 29 genes. We identified 4 distinct regulation patterns for synthesizing enzymes, as well as a distinct regulation pattern for degradations and transporting enzymes. This study charts the expression of endometrial endocannabinoid system genes across the menstrual cycle. Altered expression of genes that control endocannabinoid may allow fine control over endocannabinoid concentrations and their influence on cellular function, maturation and differentiation as the endometrium matures through the menstrual cycle.

## Introduction

Endocannabinoids are small lipid-based molecules that play critical roles in regulating cellular functions. The most well characterised endocannabinoids are anandamide (AEA) and 2-arachdonoylglycerol (2-AG). Their production, concentration and cellular influence is mediated by a complex interaction of proteins that regulate their synthesis, transport, membrane binding, trafficking and degradation^1^. Collectively endocannabinoids and these proteins are termed the endocannabinoid system (ECS)^2^. Altered expression of any ECS component can lead to variation in endocannabinoid activity and alter their impact of cellular function.

Synthesis and degradation of AEA involves a number of enzymes. Four routes for AEA synthesis from NAPE have been reported and the most widely accepted pathway is the single-step, direct synthesis by the enzyme NAPE-PLD^3–6^. The remaining three pathways are two-step processes involving phospholipase C (PLC) and the protein tyrosine phosphatase N22 (PTPN22)^7,8^, phospholipase A2 (PLA2) and 2-lyso-phospholipase D (LysoPLD)^9,10^, and α/β hydrolase 4 (ABHD4) and glycerophosphodiester phosphodiesterase 1 (GDE1)^11,12^. Fatty acid amide hydrolase (FAAH) is the main enzyme responsible for degradation of AEA^13,14^. In addition AEA is subjected to oxygenation by a number of enzymes including cyclooxygenase-2 (COX-2)^15,16^, 5-, 12- and 15-lipoxygenase (5-/12-/15-LOX)^17,18^, and several cytochrome P450 monooxygenases (P450s) including CYP3A4, CYP4F2, CYP4X1, and CYP2D6^19,20^. Two two-step synthetic pathways have been identified for the synthesis of 2-AG, and involve PLC^21^ followed by *sn*-1-diacylglycerol lipase (DAGL), or phospholipase A1 (PLA1) followed by lyso phospholipase C (lyso-PLC)^22,23^. The metabolism of 2-AG involves several enzymes including the main degrading enzyme monoacylglycerol lipase (MAGL)^21,24^, FAAH, ABHD6 and ABHD12^25^, COX-2 and LOXs^16,26^.

Endocannabinoids are found in many organs and tissues including the endometrium and influence cell migration^27,28^, proliferation^29^, survival^30^ inflammation^31,32^ and cellular differentiation^33^. The endometrium lines the uterus and is comprised of epithelial glandular structures, vascularised stroma and infiltrating immune cells. It is unique in that it undergoes cyclical growth, regeneration and shedding each month. Endometrial maturation during the menstrual cycle is controlled by female sex hormones, but also requires tightly regulated cellular responses to facilitate the cellular and structural changes. The proliferative phase is dominated by estrogen-driven cellular proliferation, vascularisation and immune stimulation while the secretory phase is dominated by progesterone mediated stromal cell decidualisation, vascular remodelling and immune cell modulation^34^. Regeneration of the endometrium following menses is mediated through adult stem and progenitor cells located in the basalis layer of the endometrium that proliferate, mature and differentiate across the menstrual cycle^35,36^. The consistent cyclical shedding and regrowth creates the monthly potential for errors in cell replication that may underlie both permanent, or transient endometrial abnormalities.

Endocannabinoids are abundantly expressed in the endometrium and influence endometrial cellular function. Synthetic endocannabinoid, methandamide induces endometrial stromal cell migration^27^, and AEA impairs both cellular proliferation and differentiation of an immortalised endometrial stromal cell line (St-T1b) and human decidual fibroblast from placenta, suggesting a crucial role in decidualisation^37–39^. AEA was also shown to influence migration in the immortalized human endometrial epithelial cell line HEC-1B^28^. Variations in concentrations of endocannabinoids may influence cellular proliferation and differentiation as the menstrual cycle progresses and the endometrium matures. Genes within the ECS are increasingly being recognised as targets to modulate endocannabinoid activity for multiple reproductive diseases including endometriosis, miscarriage, ectopic pregnancy, pre-eclampsia and endometrial cancer^39,40^. How these are altered across the menstrual cycle is not yet clear.

We hypothesised that the ECS plays a role in the cellular function of the endometrium, that they are regulated across the menstrual cycle and that this may have implications for how the endometrium matures. The goal of this study was to define an a priori set of ECS genes from the literature, analyse their mRNA expression in human endometrial tissue and determine variations in their expression across the menstrual cycle.

## Results

### ECS gene expression in the endometrium

**Figure 1:**
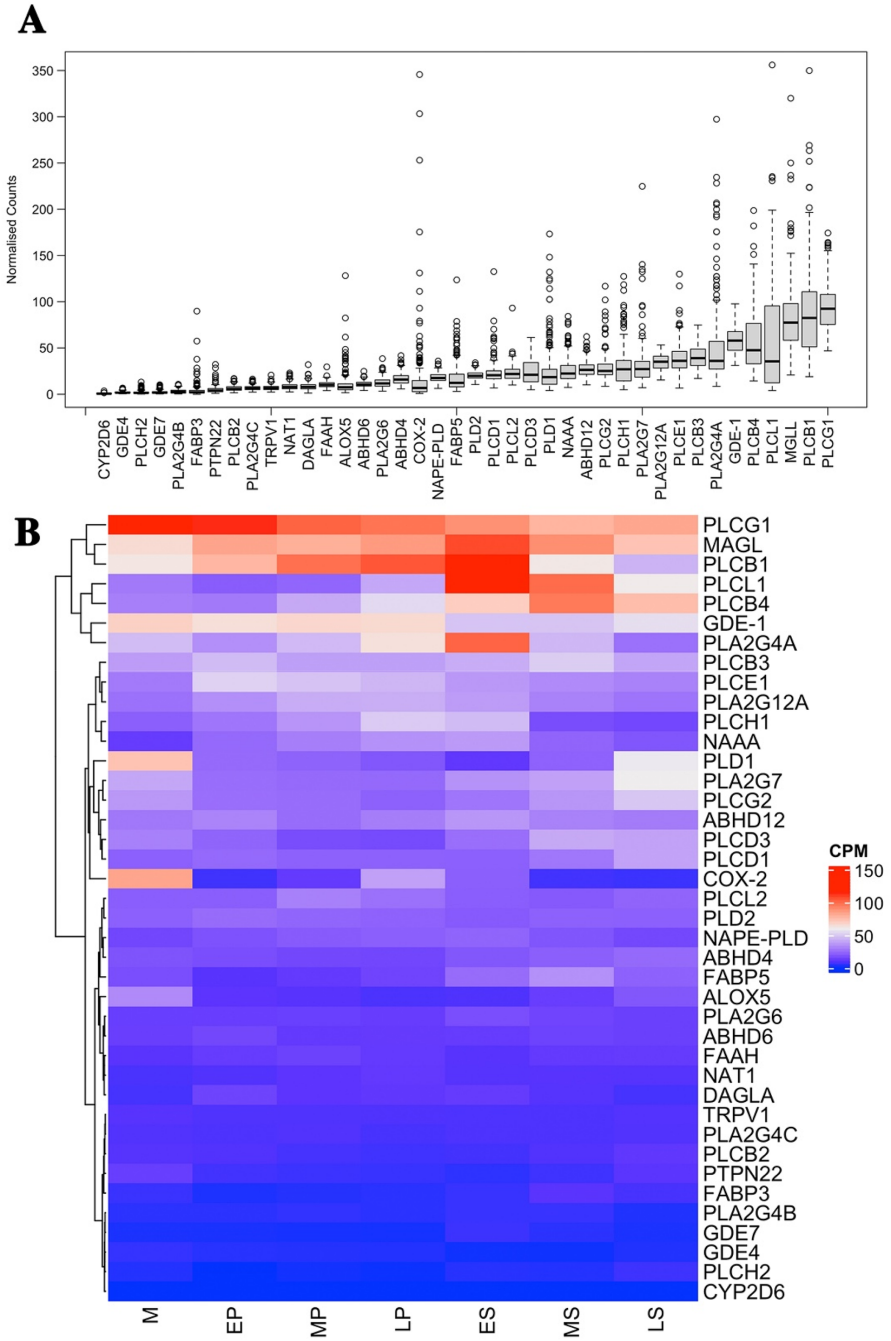
Gene expression of ECS genes in endometrium across the menstrual cycle. (**A**) Using 206 endometrial samples we found that of the 70 genes identified through the literature to function as part of ECS, 40 were expressed in at least 90% of the samples. The level of expression of each gene varied significantly between individuals and mean expression varied between genes. (**B**) A heat map analysis identifying the relative expression (cpm) of each gene across the menstrual cycle. The stage variation identified for some of these genes could be explained in part by differences that occurred in expression across the menstrual cycle. M = Menstrual, EP = Early proliferative, MP = Mid proliferative, LP = Late proliferative, ES = Early secretory, MS = Mid secretory, LS = Late secretory.

Of the 70 genes selected from a priori list for examination we found only 1 gene with no discernible expression in any endometrial samples (*PLA2G2E*), 64 were expressed in at least 1 sample with > 10 counts, 61 were expressed in 3 or more samples, 40 were expressed in greater than 90% of all samples (Supplementary Table S1). Synthesizing enzymes accounted for 45 of the investigated genes, 29 (64.4%) of which were expressed in > 90% of samples. Endocannabinoid degrading enzyme genes accounted for 12 of the investigated genes, of which 8 (66.7%) were expressed in > 90% of samples. Genes encoding transport proteins accounted for 7 of the investigated genes of which 2 were consistently expressed in > 90% of the samples. Finally, 6 genes for endocannabinoid membrane receptors were investigated and only one of these was expressed in > 90% of the samples. We focused the subsequent analysis on these 40 genes expressed in > 90% of the samples and their expression patterns across the menstrual cycle. The relative mean expression of each gene showed significant variation (Fig. 1A) and a heatmap analysis charting gene expression against the menstrual cycle indicated a large degree of this variability could be explained by changes associated with cycle stage (Fig. 1B). Five most expressed genes across the menstrual cycle are listed (Table 1).

**Table 1:** List of 5 most expressed genes across the menstrual cycle.

Menstrual	Early proliferative	Mid proliferative	Late proliferative	Early secretory	Mid secretory	Late secretory
PLCG1	PLCG1	PLCG1	PLCB1	PLCB1	PLCL1	PLCG1
COX-2	MAGL	PLCB1	PLCG1	PLCL1	PLCB4	PLCB4
PLD1	PLCB1	MAGL	MAGL	MAGL	MAGL	MAGL
GDE-1	GDE-1	GDE-1	GDE-1	PLA2G4A	PLCG1	PLCL1
MAGL	PLCE1	PLCE1	PLA2G4A	PLCG1	PLCB1	PLA2G7

5 highly expressed genes for each of the 7 menstrual stages with the most expressed gene at the top. The majority of the genes listed encode for synthesising enzymes of the endocannabinoid system with two exceptions of COX-2 and MAGL which encode for degrading enzymes.

### ECS gene expression in women with and without endometriosis

Endometriosis is one of the most common endometrial pathologies occurring in 1 in 9 reproductive age women^41,42^ with suspected endometriosis the most common indication for surgery in this cohort. To determine whether endometrial pathologies, such as endometriosis, could influence endometrial ESC gene expression, we assessed the differences between women with and without endometriosis. Of the 206 samples collected 143 were confirmed endometriosis cases from visual inspection of the pelvis at laparoscopy and 63 were without evidence of endometriosis. Using the a priori set of selected genes and comparing the expression between women with and without endometriosis, no genes showed evidence of significant difference of expression when adjusted for multiple testing either compared together and taking into account menstrual cycle as a co-variate (Supplementary Table S2), or when comparing each cycle stage individually (Supplementary Table S3–S8). Previous studies on gene expression differences in these samples showed no genome-wide significance differences between women with and without endometriosis^43,44^. In subsequent analysis of expression across the menstrual stage therefore, we assessed all samples together, regardless of endometriosis status.

### Gene expression of enzymes responsible for endocannabinoid production

We performed 6 comparisons of gene expression between the 7 consecutive stages of the menstrual cycles, adjusting the p value to account for multiple testing of both multiple genes and menstrual cycle stage tests (Supplementary Table S9–S14). We identified a significant difference in 21 genes between the 7 consecutive stages of the menstrual cycle. Using a heatmap analysis we compared the expression of these genes across all stages and grouped those that showed similar levels of expression and pattern of regulation**,** identifying four distinct patterns of interest.

**Figure 2:**
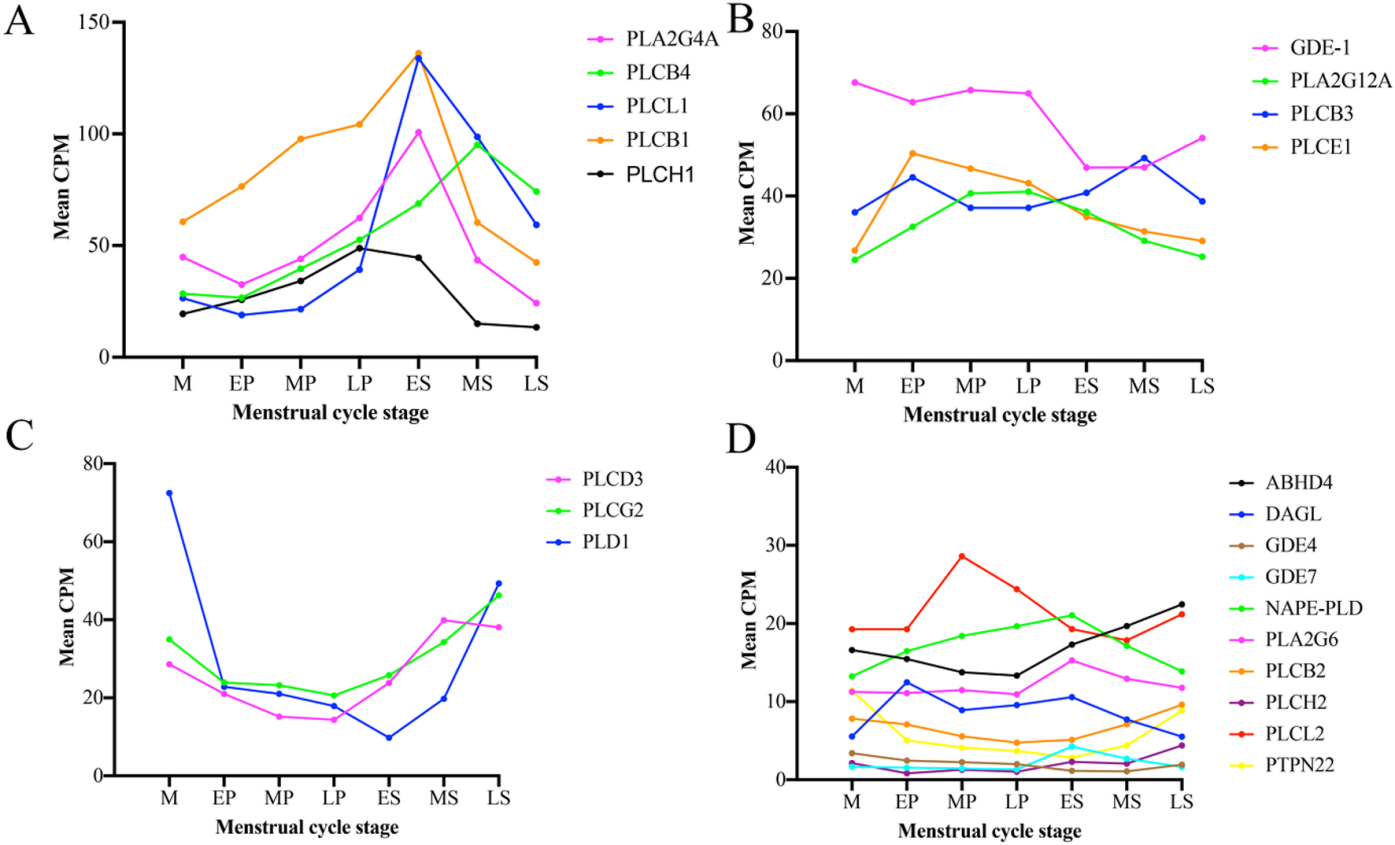
The expression profile of the four distinct groups of endocannabinoid synthesizing enzymes. (**A**) Group 1 showed high gene expression (≈ 50–150 CPM) that increased across the proliferative stage and peaked during the early secretory to mid secretory stage. (**B**) The second group was characterised by median gene expression (≈ 20–80 CPM) that generally increased from the menstrual to early proliferative stage and then showed a gradual decline. (**C**) This group was characterised by a low- medium expression (≈ 10–80 CPM) that decreased in the early proliferative stage and subsequently increased in the secretory stage. (**D**) The fourth group was characterised by low gene expression (≈ 1–30 CPM) that showed only small changes across the stages. M = Menstrual, EP = Early proliferative, MP = Mid proliferative, LP = Late proliferative, ES = Early secretory, MS = Mid secretory, LS = Late secretory.

The first group contained five genes with a high median gene expression that increased consistently across the menstrual cycle from the EP stage and peaked in either the ES (*PLA2G4A, PLCL1, PLCB1*) or MS stage (*PLCB4*) followed by a decline in expression in the LS (Fig. 2A). Significant increases were observed for *PLCL1* between the MP to LP stage (*p* = 0.004) and the LP to ES stage (*p* = 8.28 × 10^−15^), but was subsequently down regulated in the MS stage (*p* = 0.007). There was a significant increase in *PLCB4* from the LP to ES stage (*p* = 0.007) with a further increase from the ES to MS stage (*p* = 0.003). A significant difference was recorded in the transition from the ES to MS stage for *PLCB1* (*p* = 1.31 × 10^−11^). A significant decrease was observed for *PLA2G4A* in the transition from the MS stage to the LS stage (*p* = 1.31 × 10^−11^).

The second group of genes was characterised by a moderate mean expression with a gradual decrease across the cycle after initially peaking during the EP stage. An exception was observed with *PLCB3* that showed a consistent expression across the cycle peaking in the MS stage (Fig. 2B). Significant increases in expression were observed during the transition from the M to EP stage for *PLCE1* (*p* = 0.004) and *PLA2G12A* (*p* = 0.001) that also showed a significant decrease from the LP to ES (*p* = 0.007), as well as the ES to MS stage (*p* = 1.44 × 10^−8^). Significant decreases were also observed for *GDE1* from the LP to ES stage (*p* = 7.81 × 10^−8^) and for *PLCB3* in transition from the MS to LS stage (*p* = 0.003).

The third group was characterised by medium mean expression and that decreased directly after the menstrual stage and then subsequently increased again after the ES stage (Fig. 2C). Significant decreases in *PLD1* were observed in the transition from the M to EP stage (*p* = 0.0005) and again from the LP to ES transition (*p* = 0.002). A significant increase was observed for *PLCG2* (*p* = 0.005) and *PLCD3* (*p* = 8.01 × 10^−5^) during the LP to ES transition. *PLD1* began to increase in the MS stage (*p* = 0.0003) continuing into the LS stage (*p* = 2.8 × 10^−8^). Both *PLCG2* (*p* = 0.005) and *PLCD3* (*p* = 1.44 × 10^−8^) continued their increase during the early to mid-secretory stage.

The final group was characterised by low mean expression that showed multiple and varied expression changes across the EP to ES stage (Fig. 2D). Significant differences for the individual genes were observed between the M to EP stage for *DAGL* that was increased (*p* = 0.003) and *PTPN22* that was decreased (*p* = 0.003). There was minimal regulation across the proliferative stage, however in the transition from the LP to ES stage there was a decrease in *GDE4* (*p* = 5.48 × 10^−5^), *PLCL2* (*p* = 0.007) and an increase in *GDE7* (*p* = 2.93 × 10^−11^), *ABHD4* (*p* = 0.002), *PLCH2* (*p* = 0.0001) and *PLA2G6* (*p* = 0.007). Significant changes also occurred in the ES to MS stage with a reduction in *PLCH2* (*p* = 2.19 × 10^−16^), *DAGL* (*p* = 0.0004), *GDE7* (*p* = 0.0009) and an increase in *PLCB2* (*p* = 0.002) and *PTPN22* (*p* = 0.001). *NAPE-PLD* decreased from the MS to LS (*p* = 0.003). Finally, *PLCH2* (*p* = 0.003), *PTPN22* (*p* = 0.0002) and *GDE4* (*p* = 0.003) were all significantly increased in LS compared to the MS stage.

### Gene expression encoding endocannabinoid transporters

**Figure 3:**
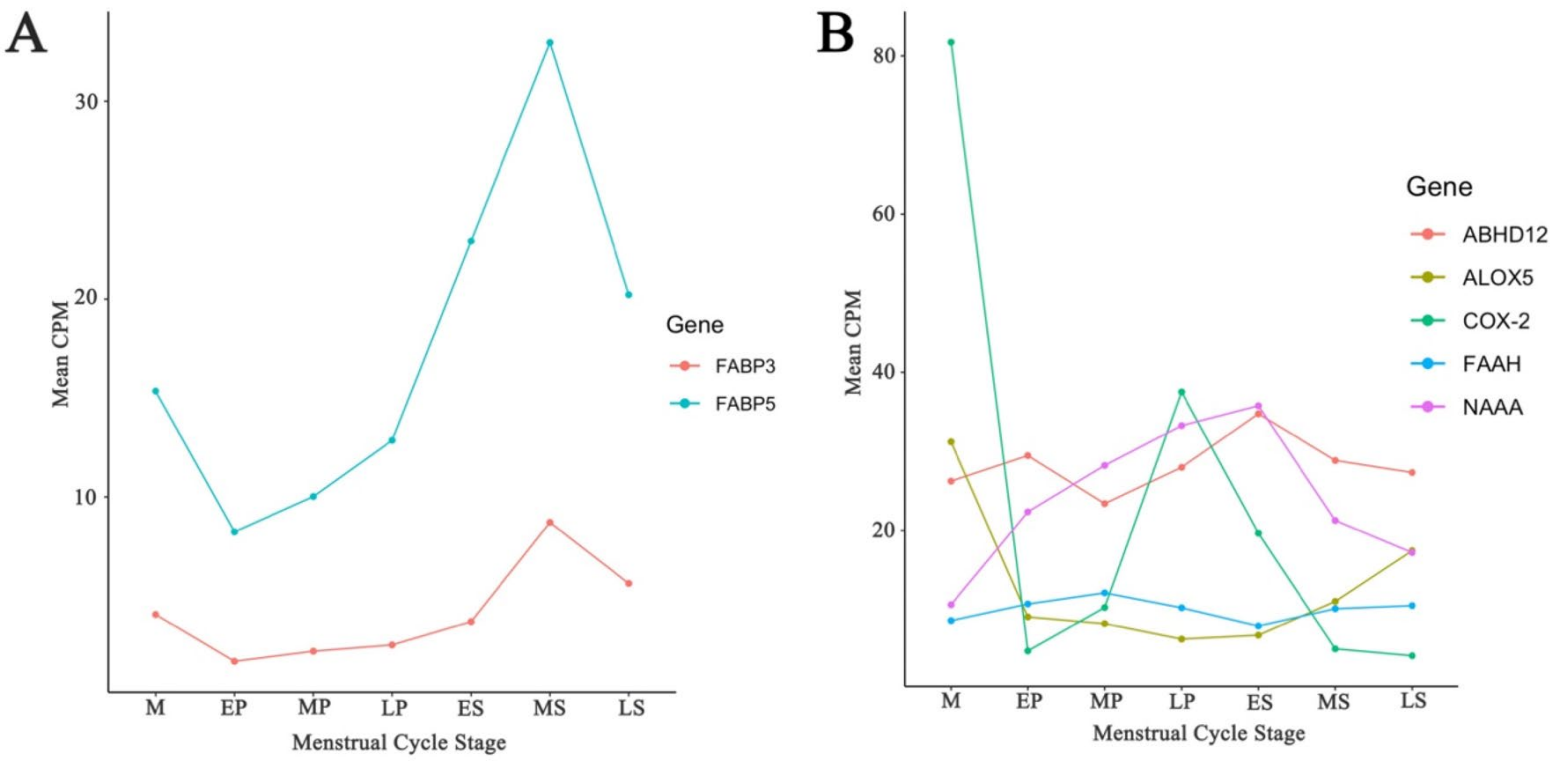
The expression profile of genes encoding ECS transport and degrading proteins. Transcription expression that showed a significant difference at any stage in the menstrual cycle were plotted against cycle stage. (**A**) For the transporting proteins moderate expression was observed for FABP5 and FABP3 that was significantly increased across the menstrual cycle. (**B**) ECS degrading enzymes had a moderate to low expression that were most commonly altered between the menstrual and early proliferative stage, or during the period from the late proliferative to mid secretory stage. Two genes ABHD12 and NAAA showed a consistent increase over the proliferative stage, after which there was decrease in expression. M = Menstrual, EP = Early proliferative, MP = Mid proliferative, LP = Late proliferative, ES = Early secretory, MS = Mid secretory, LS = Late secretory.

We identified 7 genes known to encode fatty acid-binding proteins (FABPs) that transport endocannabinoids intracellularly. The largest changes were observed for *FABP5* which was significantly upregulated from the LP to ES stage (*p* = 0.002) (Supplementary Table S12) with a subsequent down regulation from the MS to LS stage (*p* = 0.01) (Fig. 3A) (Supplementary Table S14). *FABP3* was significantly upregulated in the transition from the ES to MS stage (*p* = 0.0005) (Supplementary Table S13).

### Gene expression of enzymes responsible for degradation of endocannabinoids

We identified expression of 8 of the 12 genes related to endocannabinoid degradation in endometrial tissue. Of these *ALOX5* (*p* = 0.0005) and *COX-2* (*p* = 0.008) were significantly down regulated and *NAAA* significantly upregulated in the transition from the M to the EP stage (*p* = 0.008) (Fig. 3B) (Supplementary Table S9). In the transition from the LP to ES stage, *FAAH* was decreased (*p* = 0.002) and *ABHD12* was increased (*p* = 0.001) (Fig. 3B) (Supplementary Table S12). In contrast in the ES to MS stage *FAAH* was upregulated (*p* = 0.0003) and *ABHD12* downregulated (*p* = 0.004) (Supplementary Table S13). Other significant changes from the ES to the MS stage included; *NAAA* (*p* = 5.04 × 10^−7^) and *COX-2* (*p* = 1.15 × 10^−6^) that were downregulated and *ALOX5* (*p* = 0.0002) that was upregulated.

### Gene expression of endocannabinoid receptors

**Figure 4:**
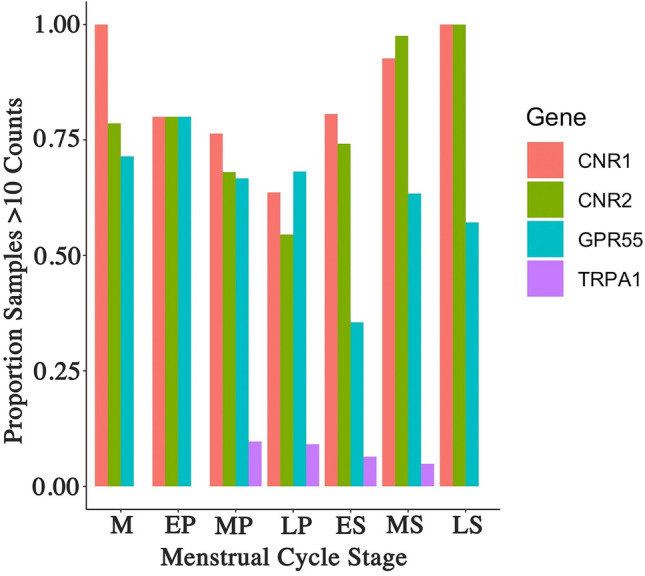
Logistic regression analysis of endocannabinoid receptor. Four of the five endocannabinoid receptor did not show consistent expression above > 90% in all samples. A logistic regression analysis examining the percentage of samples displaying a positive expression showed significant variation across the menstrual cycle for CNR1, CNR2 and GPR55. Only a small number of samples expressed TRPA1 and this was limited to the period between the mid proliferative to mid secretory stage. M = Menstrual, EP = Early proliferative, MP = Mid proliferative, LP = Late proliferative, ES = Early secretory, MS = Mid secretory, LS = Late secretory.

We assessed gene expression of 6 ECS membrane receptors across the menstrual cycle and between endometriosis status. Of these 6 genes we found that only one, *TRPV1* was expressed in > 90% of the samples. It had a low but consistent expression and was not significantly regulated across the menstrual cycle or influenced by endometriosis status. The expression of all other membrane receptors occurred in < 90% of the samples. This suggests either a consistently low expression below read depth, or a binary on/off regulation. Based on the potential for binary regulation we determined the percentage of samples expressing membranes receptor genes in each menstrual stage (Fig. 4). For both *CNR1* and *CNR2* the number of samples with positive expression was between 70 and 100% for the various stages of the cycle, decreasing through the proliferative stage with a subsequent rise during the secretory stage. The changes however did not reach significance. *GPR55* expression occurred in approximately 70% of samples, also decreasing through the proliferative stage reaching a minimum during the ES stage and subsequently increasing during the MS to LS stages. *TRPA1* expression was observed only between the MP and MS stage and only in a low number of samples. Statistical analysis revealed there was no significant difference for any endocannabinoid receptor across the different stages of the menstrual cycle. Furthermore, no difference was observed in the percentage of samples expressing any of the membrane receptors when comparing endometriosis status and adjusting for menstrual cycle phase.

### Correlation analysis with microarray

Microarray data was available on a subset of samples (n = 161). 35 of the 40 genes were detected in at least 50% of the microarray samples and included in the analysis to ensure sufficient pairwise comparisons to estimate an accurate correlation. Using the ‘cor.test' function in R we estimated the Pearson correlation between normalized and batch corrected counts from RNA-Seq (log2-CPM) and microarray (log2-normalised signal) for each gene across the 161 samples. All 35 genes detected were positively correlated with a mean correlation value of 0.62 confirming a robust measurement of gene expression using the RNA-seq technique, despite the differences between microarray and RNA-seq including the inability of microarray to measure all transcript variations and different detection thresholds (Supplementary Table S15).

## Discussion

Endocannabinoids, naturally synthesized lipids are produced in abundance in the female reproductive tract^45^. They are present in normal endometrium and significantly influence cellular maturation and function^46^. Modulation of the ECS system occurs through enzymes regulating production, transport and degradation of the endocannabinoid signaling molecules. Understanding enzyme expression may provide insights into how endocannabinoid levels are modulated in endometrium. Utilizing genome-wide gene expression data of endometrial samples we performed a comprehensive analysis of ECS gene expression during the menstrual cycle. We identified significant regulation across the menstrual cycle for many genes and identified distinct patterns of regulation that strongly support alteration in endocannabinoid activity across the menstrual cycle that could be both a consequence of, or contribution to the changes that occur during cyclical endometrial maturation.

Enzymes capable of synthesizing endocannabinoid production were the most abundant in the endometrium, and also showed the greatest level of menstrual cycle regulation. The majority of gene expression changes occurred during the transition from the late proliferative to early secretory stage and continued across the secretory stage. The transition from proliferative to secretory stage is characterized by ovulation and an increase in the production of progesterone. Progesterone levels also fluctuate during the secretory stage by increasing up to the mid secretory stage and decreasing during the late secretory stage^34^. Gene expression changes across the cycle suggest an important role of steroid hormones in ECS modulation, in particular progesterone. Previous studies have reported progesterone is involved in the maintenance of endocannabinoid levels of the endometrium^47^. Progesterone was found to induce CB1 expression in endometrial stromal cells^31^, and to activate FAAH promoter decreasing AEA levels in human T lymphocytes^48^. In mice, progesterone has been shown to down-regulate uterine NAPE-PLD expression, possibly leading to a decrease in AEA levels^49^. Although in contrast, one study reported no correlation between plasma levels of AEA and progesterone in normal cycling women^50^.

The most abundant endocannabinoids in the endometrium, AEA and 2-AG, are predominantly synthesized via NAPE-PLD and DAGL respectively^1^, representing two of the most important enzymes in the ECS. We found both enzymes were downregulated during the transition from menstrual to early proliferative stage and then subsequently upregulated from early to mid-secretory and again from the mid to late secretory stage. This suggests an increased capacity for endocannabinoid production as the menstrual cycle progresses. This increased capacity may not be linked to progesterone as it decreases towards the end of the cycle, a period where endocannabinoid production capacity is still increasing. A previous study reported NAPE-PLD immunoreactivity was increased in the menstrual, early proliferative and late secretory glands with its lowest levels in the early secretory phase^51^. In a mouse model progesterone was reported to decrease NAPE-PLD expression^49^. The increase in these enzymes and the potential increase in the endocannabinoids may have implications for cellular function and differentiation during the menstrual cycle in humans and rodents.

A number of other enzymes can regulate endocannabinoid production through alternate pathways. One such group includes the many isoforms of phospholipase C (PLC). PLCs selectively catalyze the degradation of phosphatidylinositol 4,5 bisphosphate resulting in soluble inositol-1,4,5-triphosphate (IP3) and membrane delimited 1,2 diacylglycerol (DAG)^52^. DAG is an important precursor for 2-AG^53^. We observed multiple, but distinct patterns of *PLC* regulation across the menstrual cycle, particularly during the transition from the proliferative to secretory stage. This regulation would impact DAG expression subsequently influencing 2-AG production. Whether each of these isoforms has similar efficacy in the metabolism of IP3 and the subsequent production of DAG in the endometrium is unclear from this study.

Endocannabinoids continue to exert their activity until degraded. A number of enzymes responsible for the degradation of endocannabinoids, including FAAH, MGLL and ABHD12 were also significantly downregulated during the transition from the late proliferative to early secretory stage. A down regulation of these enzymes would potentiate the ability of endocannabinoids to continue signaling. Of these enzymes FAAH is of particular interest. FAAH is a principal enzyme in the degradation of AEA and previous evidence suggests a relationship to progesterone^54,55^. FAAH has previously been implicated in the survival of endometrial stromal cells from ectopic endometriosis lesions^56^ and has received significant interest as a pharmacological target, modulating pain through both peripheral and central mechanisms^57^. Physiological concentrations of progesterone stimulated FAAH activity in human lymphocytes, decreasing AEA levels^48,54,58^, while in a mouse model progesterone was reported to decrease uterine FAAH activity^59^. Progesterone levels and FAAH expression have been correlated during the menstrual cycle^60^, in agreement with the finding that progesterone up-regulates *FAAH* gene expression^48,58^. Combined with the regulation of the *PLC*s during this period, this suggests a regulation of endocannabinoid turnover during the transition between menstrual cycle stages that is tightly regulated.

Additional factors, other than their production and inhibition, can influence endocannabinoids concentrations and their activity. Intracellular degradation relies on lipophilic endocannabinoid reaching the intracellular enzymes. One of the most significant changes we observed in this gene set was the regulation of *FABP5* across the menstrual cycle. FABP5 is a fatty acid binding protein that transports endocannabinoids through the aqueous cellular environment, preferentially directing it towards FAAH^61^. *FABP5* was also significantly downregulated during the transition from the late proliferative to the early secretory stage. A reduction in the transport towards the degrading enzyme could potentiate the presence of the endocannabinoids during this period and may contribute to cellular and inflammatory changes^34,62^ that enables the regeneration of the endometrium in the early and mid-secretory phase in non-conceptive cycles.

Although we included an extensive list of genes encoding endocannabinoid receptors, including *CNR1, CNR2, TRPV1, TRPA1, GPR55*, and *GPR119* we found that only one of these (*TRPV1*) was consistently expressed in > 90% of the samples. Both CNR1 and CNR2 were previously shown to be expressed in the endometrium with variation in expression between women with and without endometriosis^31^. We found expression in the majority of samples, but not sufficient to reach the > 90% cut off. Analysis of gene expression for the remaining receptors as a binary variable found changes across the menstrual cycle. Although we did not find any that reached significance after multiple testing, this may be related to the power of the sample size, or the complex milieu of individual cell types and maturing cell states that exist within the endometrium at any point in time. The results showed non-significant changes in number of samples expressing *CNR1, CNR2* and *GPR55* during the late proliferative to early secretory period. While CB1 receptor (the protein encoded by the *CNR 1* gene) was found to inhibit human decidualization and stimulates apoptosis^38^, studies on CB1 expression in the epithelial glands found no significant regulation across the menstrual cycle^51^, and CB2 expression in stromal cells was similar between the proliferative and the secretory phase^30^. Previous studies have also reported that progesterone exerted minimal effects on CB1 expression in lymphocytes^48,54,58^. It is therefore likely that the lack of significant changes in the expression of these receptors across samples and across the menstrual cycle indicates they may not be significantly regulated by reproductive hormones in endometrial tissue. The ability of these genes to be switched on or off may reflect the selective use in patients, depended on local requirements and provide the ability to fine tune the activity of the ECS.

From our study cohort, 143 (69.4%) were diagnosed with endometriosis. Previous studies on the ECS in women with endometriosis have shown conflicting results. One reported no significant difference in *NAPE-PLD* and *FAAH* expression in the endometrium of patients compared to women without endometriosis throughout the menstrual cycle^63^. Another study reported no difference in CB1 protein expression during the proliferative phase between patients with and without endometriosis^63^, whereas lower levels of *CNR1* and the protein CB1 have been reported in endometrial tissue from women with endometriosis compared to controls regardless of the cycle phase^31^. No difference in *TRPV1* expression between women with and without endometriosis throughout the menstrual cycle has also been reported^63^. In this current study, when accounting for menstrual stage and multiple testing, we found no significant difference in any of the ECS genes investigated between women with and without endometriosis. Our data therefore correlates with previously published data that shows the ECS is not significantly dysregulated in eutopic endometrial tissue from patients with endometriosis.

This is the largest number of endometrial samples utilized to investigate gene expression of the ECS through menstrual cycle, and samples were assigned to detailed 7 menstrual stages following the Noyes criteria. However, the distribution of samples across the menstrual stages varied and the results should be interpreted with caution when the sample sizes for menstrual stages were small. While we were able to analyze the majority of the ECS at the gene level with RNA-seq data, we are yet to translate this to protein. Difference in gene and protein expression are commonly identified, which is likely to replicated in the ECS system. We aim to generate a truly representative picture of the ECS at the protein level once sufficient sample and resources have been collected.

In summary, we have utilized genome wide gene expression to investigate a specific predefined gene set based on their previously identified involvement in the ECS. Targeted analysis of a defined gene set will reduce the burden of multiple testing in gene expression data of modest size providing the potential to uncover subtle variations. We detected differences in the gene expression across the menstrual cycle that are most pronounced during the transition from the proliferative to secretory stage and reflect a potential to dynamically modulate endocannabinoid concentrations during this period. As the activity of endocannabinoids are rapid and short-lived, we speculate that the altered regulation of expression for many components of the ECS across the menstrual cycle provides a quick acting, fine tuning potential to modulate endocannabinoid expression as needed, particularly during the transition period in which the endometrium undergoes major structural rearrangement. Of particular interest, we noted the large changes in a number of PLC enzymes, and the significant variation in *FAAH* and *FABP5* that work in concert to deactivate endocannabinoid activity and are already receiving considerable attention as drug targets for pain, inflammation and cancer^64,65^. The important ability of endocannabinoid receptors to be regulated may also hold particular interest for the potential of selective targeting of patients and the personalisation of medication.

## Methods

### Sample collection

Samples were collected as described previously (n = 206)^43^ and inclusion criteria were European ancestry and within reproductive age (18–49, mean = 31.85). Exclusion criteria were the use of hormonal medication within 3 months prior to surgery, abnormalities in histopathological examination, ambiguous disease status or menstrual cycle stage. Informed consent was obtained from all subjects. A histological assessment was performed on formalin fixed paraffin embedded tissue with each sample assigned to 1 of 7 possible menstrual stages following the Noyes criteria^66^: menstrual (M) (n = 14), early proliferative (EP) (n = 5), mid proliferative (MP) (n = 72), late proliferative (LP) (n = 22), early secretory (ES) (n = 31), mid secretory (MS) (n = 41) and late secretory (LS) (n = 21) (Supplementary Fig. 1). If endometriosis was observed during laparoscopic surgery lesions were excised and sent to pathology for histopathological confirmation (n = 143 endometriosis cases and n = 63 non-endometriosis controls). The study was approved by the Human Research Ethics Committee (HREC) of the Royal Women’s Hospital (Projects 11-24 and 16-43 and the Melbourne IVF Project 05-11) and the University of Queensland HREC (2016000746), and all experiments were performed in accordance with relevant guidelines and regulations.

### RNA extraction and sequencing

Total RNA was isolated from endometrial biopsies stored in RNA*later* using the Allprep DNA/RNA Mini Kit (Qiagen, USA) as per the manufacturer’s instructions and isolated RNA samples were treated with the Turbo DNA-free kit (Thermo Fischer Scientific, USA). RNA quality was confirmed using the Agilent Bioanalyzer 2100 (Agilent Technologies, USA) with RNA integrity number (RIN) cut off values set at above 8 for inclusion and final RNA concentrations determined by the Nanodrop ND -6000 (Thermo Fisher Scientific, USA). Stranded RNA-sequencing (RNA-seq) libraries were prepared with the Illumina TruSeq Stranded Total RNA Gold protocol incorporating ribosomal depletion (Illumina, USA). The resulting libraries were pooled and sequenced with 75 bp paired-end reads on the Illumina HiSeq 4000 to a mean depth of 37,490,673 for 178 samples and with 120 bp paired-end reads on an Illumina Hi Seq 2000 (Illumina, USA) for a mean depth of 40,818,062 reads for 28 samples.

### Preparation of RNA-seq data

The quality of raw RNA-seq reads were confirmed with FastQC v0.11.7^67^ and MultiQC v1.6^68^. Low quality reads, or reads containing HiSeq Illumina adapter sequences were trimmed using Trimmomatic v0.36^69^. The resulting trimmed reads were aligned to a reference assembly (Ensembl *Homo sapiens* GRCh38 release 91) with HISAT2 v2.0.5 and the transcript assembly performed with StringTie v1.3.1^70,71^ and each read mapping to a known transcript counted. Transcript, exon and expression matrices in Fragments Per Kilobase of transcript per million mapped reads (FPKM) were determined with StringTie counts for each individual. Prior to normalisation of RNA-seq counts lowly expressed genes (counts per million (CPM) < 0.22) and expressed in < 90% of samples were removed. Gene counts were normalised for composition bias and total raw reads using TMM^72–74^ in edgeR R package v 3.22.3^75^. The normalized counts were converted to CPM and log2 transformed (log2-CPM).

### Selection of the ECS genes

A priori gene selection was performed through a literature search using the search term ‘endocannabinoid’, and combinations of ‘pathway’, ‘synthesis’, ‘metabolism’, ‘transport’ or ‘endometrium’. All genes relevant to the control of the ECS were catalogued by ENSEMBL gene expression ID and gene expression extracted from the curated database. ECS genes of interest were split into four categories based on their main function in the ECS including (i) synthesizing enzymes, (ii) receptors, (iii) transporters, and (iv) degradation enzymes. In total 70 potential genes known to play a role in the ECS were assessed (Supplementary Table 1).

### Gene expression analysis

Differential gene expression was investigated both across the menstrual cycle and in women with and without endometriosis. Only genes with CPM > 0.22 in at least 90% of samples were analysed for differential expression. Seven differential gene expressions comparisons were performed across the menstrual cycle: (i) between M and EP, (ii) between EP and MP, (iii) between MP and LP, (iv) between LP and ES, (v) between ES and MS, (vi) between MS and LS and (vii) between MP and MS. Seven differential gene expressions comparisons were performed between women with and without endometriosis: (i) including stage of menstrual cycle as a covariate, (ii) within M, (iii) within MP, (iv) within LP, (v) within ES, (vi) within MS and (vii) within LS. Batch effects (flow-cell and lane) were fitted as covariates in all models. The voom function in limma^76^ was used to transform the normalised counts to log counts per million with associated precision weights prior to linear modelling. The pairwise comparisons described above were made using the eBayes method whereby a moderated t-statistic and log-odds of differential expression is estimated for each gene for each contrast. *p* values were adjusted for the number of genes tested and for the comparison of multiple cycle stage comparisons using Benjamini–Hochberg with a significance threshold of 0.05. The data underlying this article are available on the article and in its online supplementary material.

In the case of membrane receptors where positive gene expression was consistently < 90% of the samples, we explored gene expression as a binary function of expressed/not expressed and performed a logistic regression analysis as described previously^43^ to determine if there was as significant variation in the number of samples that show a positive expression of these genes across the menstrual cycle.

## Supplementary Information

Supplementary Figure 1.

Supplementary Information 2.

Supplementary Information 3.

Supplementary Information 4.

Supplementary Information 5.

Supplementary Information 6.
