# Supplementary material for: Gene expression of the endocannabinoid system in endometrium through menstrual cycle
Source: Sci Rep. 2022 Jun 7;12:9400. doi: 10.1038/s41598-022-13488-4 (PMC9174470; doi:10.1038/s41598-022-13488-4)
Supplement: Supplementary file 1 — Supplementary Figure 1. [file 41598_2022_13488_MOESM1_ESM.docx]

**Supplementary figure 1: Hematoxylin and eosin stained histology images for 7 menstrual stages of the endometrium.** Representative histology images that were used to categorize each sample into 1 of 7 different menstrual stages based on morphological descriptions outlined in the Noyes criteria. This included samples from the **(A)** menstrual **(B),** early proliferative **(C),** mid proliferative **(D),** late proliferative **(E),** early secretory **(F),** mid secretory **(G)** and late secretory stage.
